# A model for cost analysis–application to clinical laboratory test economics using computer facilities

**DOI:** 10.1155/S1463924686000354

**Published:** 1986

**Authors:** F. R. Hindriks, W. van der Slik, A. Bosman, Chr. Frowein, H. Kamps

**Affiliations:** 1 Central Laboratory for Clinical Chemistry University Hospital Groningen Oostersingel 59 Groningen 9713 EZ The Netherlands; 2 Department of Management Science and Business Administration of the Economic Faculty University of Groningen Groningen The Netherlands; 3 Financial and Material Department University Hospital Groningen The Netherlands


Journal of Automatic Chemistry/*Journal of Clinical Laboratory Automation*, Vol. 8, No. 4 (October–December 1986), pp. 178–185

### A model for cost analysis–application to clinical laboratory test economics using computer facilities

**F. R. Hindriks, W. van der Slik,**
*Central Laboratory for Clinical Chemistry, University Hospital Groningen, Oostersingel 59, 9713 EZ Groningen, The Netherlands*

**A. Bosman, Chr. Frowein**
*Department of Management Science and Business Administration of the Economic Faculty, University of Groningen, Groningen, The Netherlands*

**and H. Kamps**
*Financial and Material Department, University Hospital Groningen, The Netherlands*

### Introduction

In the last few years, both inside and outside the clinical chemical profession, there has been a growing concern about the cost of clinical chemistry analysis. The continuing increase in the cost of health care has led to the health insurance companies paying special attention to the cost of laboratory investigations. Dutch clinical chemists discussed this problem at a recent congress [1].

Many studies have been conducted on the topic of costing problems for clinical chemistry investigations [2–12]. In The Netherlands De Vries [13] and Leijten [1 and 14] have carried out extensive examinations in this field; the latter used the input-output method for cost price calculation, showing that it is possible to define relations between different categories of cost.

In this study the cost price of clinical chemistry analysis is estimated on the grounds of data specifying the production process. These data are arranged in a generally applicable model so that the financial consequences of alternatives in the production processes in the laboratory can be quickly determined. This model is based on the 'production centres' method [15].

### Production factors and costs

In every organization, technical transformation processes take place, converting production factors into final products. An economical judgement of an organization usually takes place by means of a comparison between the production factors used and the income from the sale of the final products. In order to make such a comparison possible, the production factors used must be placed under a common denominator with the aid of the prices of the production factors. The result of this aggregation is called 'cost'. However, information is inevitably lost through aggregation and this applies in the composition of the aggregate cost. The most important stages at which information losses occur, whilst determining the cost are discussed in this paper.

(1) Production factors are used for the production of 'activities'. An activity is the output of a technical transformation process, which can be described by relating the output to the production factors used. In economics this relationship is called 'production function'. A simple production function is the following:

$$g(j) = a(1).x(1) + a(2).x(2) + \dots + a(n).x(n) \quad (1)$$

where:

$g(j)$ = the number of units of activity $j$ produced in a certain time period.

$a(i)$ = the amount of production factor $i$ ($i$ = 1 to $n$), which must be applied to produce one unit of $g$; $a(i)$ are called the 'technical coefficients'.

$x(i)$ = the amount of production factors $i$ which must be used in order to realize the production quantity $g(j)$.

An example of (1) is the production of a drug where the $x(i)$ refers to the following production factors: raw materials, laboratory accessories, labour, energy and machine time.

The concepts activity and final product are not synonymous. The set of activities can be divided into two subsets, *viz.* the subset of final products and the subset of intermediates. The final products are sold by the organizations, the intermediates are necessary to produce the final products. In order to be able to determine the cost of the final products, the cost of these intermediates must be allocated to the final products. This allocation is a form of aggregation which, as far as the execution is concerned, differs from organization to organization (see below). The cost of the intermediates are called indirect costs. The cost of the final products, before the indirect costs are included, are the direct costs.

(2) A large number of activities can be defined in every organization. However, if one wishes to take into consideration the relations between different activities, especially the ones between intermediates and final products, aggregation of activities will be necessary. Therefore certain activities, which have more or less comparable technical coefficients, are combined for the cost calculation. This is another form of aggregation.

(3) Given a production function such as (1), the cost of the production of $g(j)$ can be calculated in a simple manner, namely:

$$K(j) = \text{SUM } a(i).x(i).p(i) \quad (2)$$

where:

$K(j)$ = the cost of the production of $g(j)$.

$p(i)$ = the price per unit of the production factor applied ($i = 1$ to $n$).

The problem in calculating (2) is the assumption that a price per unit is known for every production factor. It thus denies the existence of fixed costs, where a price per unit is unknown, while these units cannot actually be bought separately.

If fixed cost are involved in the contemplation, a cost function arises as in (3):

$$K(j) = \text{SUM } a(i).x(i).p(i) + Kv \tag{3}$$

where:

$Kv$ = the fixed costs in the production of $g(j)$.

The fixed costs are an aggregate of all the production factors where it is not possible to buy a separate unit of a production factor because a price per unit is not available. In our economic system these are virtually all the important production factors, such as human labour and the fixed production factors (assets), like buildings, sites and machinery.

The appearance of fixed cost causes problems in the calculation of cost prices. Cost prices are necessary because final products are usually sold as separate items. The cost per item must therefore be known. To calculate the cost per item one can use two different approaches:

— divide $K(j)$ by $g(j)$;

— define a so-called normal quantity of production $g(r)$, calculate $K(r)$ and divide $K(r)$ by $g(r)$.

This last approach allows cost prices to be calculated before production takes place.

The aggregation procedures used for the calculation of cost and cost prices is the main cause for the differences between various cost and cost price calculation procedures. These procedures differ in the way they handle:

The prices by which the production factors used are to be multiplied to determine the cost, replacement versus historical cost with all its in-betweens.

The determination of the technical coefficients, standard cost versus averages as standards.

The allocation of the fixed cost, sunk cost or not, absorption (full) costing versus variable or direct variable costing.

In the case of absorption costing, the allocation of the indirect cost.

However, every outcome of these calculations assumes that the production method is known. In our opinion, cost price figures cannot be used to make decisions on the method of production as they are the result of a predetermined decision on which method to use. Cost figures can be used to compare different ways of production, if a common procedure is used to make the calculations. In this article such a procedure is discussed; it can also be used for the calculation of all kinds of cost prices.

### Production centres method

For the calculation of cost and cost prices the 'production centres' method is often used. This method is important for two reasons.

In the first place, the method defines cost centres, generally aggregates of activities, and relations between these cost centres. Two different kind of cost centres are distinguished, those for the intermediates and those for the final products. The relationships are defined between the intermediates cost centres and between these and those for the final products. There are no relations between the cost centres of the final products.

Secondly the costs are imputed using as input the so-called cost sorts. Cost sorts are a subdivision of the production factors used and defined in monetary terms, see Worth [6].

A system of equations for the cost calculation, for $j = 1$ to $m$ cost centres and $i = 1$ to $n$ cost sorts, is (for detailed explanation see the Appendix):

$$k \,.\, D = kr \tag{6}$$

where:

$k$ = a $(1 \times n)$ vector of cost sorts.

D = a $(n \times m)$ matrix of percentages.

kr = a $(1 \times m)$ vector of imputed costs per cost centre.

Transformation of $k$ into a $(n \times n)$ diagonal matrix $K$ results in:

$$K \,.\, D = KS \tag{7}$$

$KS$ is a $(n \times m)$ matrix, which is known as the 'cost distribution table'. In $KS$ the cost sorts are distributed over the cost centres. In the intermediate cost centres the indirect costs are specified. As has already been stated, indirect costs must be allocated to the cost centres of the final products. In the production centres method this is done as follows (again, for a detailed explanation see the Appendix):

$$kr = (E - F) \,.\, kt \tag{10}$$

where:

$kt$ = a $(m \times 1)$ vector of the total cost per cost centre.
$E$ = a $(m \times m)$ unit matrix.
$F$ = a $(m \times m)$ matrix of percentages specifying the deliveries between cost centres.

If $(E - F)$ satisfies a number of conditions, among others being non-singular, then the inverse can be calculated. With the aid of $kr$ (see [6]), the total costs of each of the final products can be calculated as follows:

$$kt = kr \,.\, (E - F)^{-1} \qquad (11)$$

The production centres method does not use equation (11), since the predefined $F$ specifies a triangular matrix, which can be solved row by row and therefore does not require the inversion of $(E - F)$. However, a disadvantage of this triangular matrix is that no 'backward deliveries' are possible. This condition may be a restriction, particularly in complex organizations. If computer facilities are available, this restriction can be overcome by the use of equation (11).

There are a number of objections concerning the way in which the production centres method approaches and solves the problems of aggregation. In this article only one objection will be dealt with, namely the method of aggregation which takes place during the specification of the cost centres. The choice of the cost centre usually takes place on the grounds of either organizational or functional specifications. This means that, in the hospital system, departments such as the clincial chemistry laboratory, pharmacy, administration, technical services and management are considered as cost centres producing intermediates, and the in- and out-patient clinics as cost centres producing final products. The problem is that too great an aggregation of activities has taken place. The relationship between the cost centre and its associated activities can no longer be established, making it impossible for those who are responsible for producing activities, to specify a relationship between the production of activities and the cost sorts within the cost centre.

It is also apparent that in equation (7) such a relationship is absent. The cost sorts are divided in a certain way, however the origin of the divisions and the relation with the cost sorts are not further specified. To be able to do so it is necessary to form a relationship between activities with a cost centre, because those who are responsible for the production of the activities can also be confronted with consequences in the form of cost. One method which is capable of solving this has been developed by Bosman [15] at the University of Groningen. This method is described in the next section.

### Cost structure model

Costs are aggregated amounts of production factors used, multiplied by the prices of these production factors. Many problems arise in the imputation of cost, because they cannot be disaggregated into the two components mentioned: applied production factors and prices.

Firstly, in order to be able to set up the cost structure model described here, disaggregation is necessary before a cost calculation can be carried out. This results in a detailed list of activities and cost sorts, which provides a great deal of information which is also applicable to the cost calculation in analysing the production process of activities. The cost of a certain activity, given a certain production method, consists of a vector of applied production factors necessary for the activities. Assuming $i$ cost sorts and $j$ activities, then, with the aid of technical coefficients $a(ij)$, the cost sorts ensuing from the activities may be determined, and subsequently the cost and cost prices for each individual activity. See the following equation:

$$A \,.\, g = b \qquad (12)$$

where:

$A$ = is a matrix of technical coefficients.
$g$ = is a vector of the quantities of production.
$b$ = is a vector of the applied $i$ production factors, or cost sorts in a physical specification.

Multiplying $b$ with the prices of the production factors involved, produces the vector $kr$ in equation (6). The direct imputed cost in monetary terms, see $kr$ in equation (6), results from:

$$A \,.\, G = B \qquad (13)$$

$$p \,.\, B = \mathrm{k}r \qquad (14)$$

where:

$G$ = is a diagonal matrix of $g$.
$B$ = is a distribution table in the form of physical cost.
$p$ = is the vector of prices of the production factors.

A cost price $kp$ based on the directly imputed cost of activities results from:

$$p \,.\, A = \mathrm{k}p \qquad (15)$$

The term 'cost price' is incorrect in this situation since in equation (15) the cost of the intermediates has not been allocated.

In the cost structure model, the disaggregation of cost by introducing intermediates can be solved in different ways. One method which may be used is the input-output method, and this was employed by van Halem [17] for a heart centre at a university hospital and by Leijten [1 and 14] for use in the clinical chemistry laboratory. Since $kr$ is known, the procedure defined in equation (11) can also be used. For another procedure, see Bosman and Bouma [15].

The assumption which lies behind the equations (12) to (15) is, as already stated, that a vector $p$ is known with prices per unit of the production factors, so in other words, fixed costs do not exist. Since fixed costs are generally the rule, rather than the exception, this is definitely an unusual assumption. The problem of the fixed cost is solved in the production centres method by employing the concept of a normal quantity per cost centre, whereby the fixed cost per unit of an activity can be determined. A similar procedure is used in the cost structure model. In this procedure, a normal capacity per fixed production factor is determined, instead of assigning a certain value to the vector $g$. The normal capacity can be determined by considering several activities as occurring simultaneously. Using normal capacity as denominator has the advantage that the relation between a fixed production factor and cost centres can be specified flexibly. If a normal capacity per production factor is known, then, with the aid of the fixed cost of that production factor, a price per unit of the production factor can be calculated.

The objective in setting up and applying a cost calculation system on the basis of precalculation (which can be checked on the basis of postcalculation) is to make it possible, for those who are responsible for the production of activities, to calculate the consequences of other factors: production methods, personnel and instrument capacities, production sequences; briefly, alternatives in the execution of production. Cost, as such, should be considered as the result of the method of execution of production, and as a management tool to test alternatives. Management decisions can be supported and tested in this manner. It is also important that there is an administrative system available which can calculate the prices of production factors, the tariffs for the activities supplied, and establish the relationship between the activities. The cost and cost prices of activities are dependent on the latter two factors.

Briefly summarized, the setting up of this system of cost price calculation consists of the following steps:

(1) Standardization of activities (cost centres) and production factors (cost sorts).

(2) Ascertaining the production quantities (vector $g$) for the various production centres (work stations).

(3) Determining the technical coefficients (supplies a matrix $A$ where the technical coefficients represent the relationship between the activities and the production factors) used.

(4) Establishing the normal capacity per fixed production factor, with this capacity a price per unit of the fixed production factor can be calculated.

(5) Calculating the cost and cost prices with the aid of computer facilities.

### The construction of the model

The first step in the construction of the cost structure model is to standardize the activities and production factors. In our laboratory several hundred different types of analyses are available to facilitate the health service, and a multitude of production factors are employed.

The analysis types are therefore divided into sublaboratory groups and subsequently split up according to work station. In the first instance 35 types of activities are defined in the authors' laboratory in this way. More activities may be differentiated, depending on the calculation programme used as at a latter stage. It is advisable to arrange the analysis types in such a way, that, as far as the cost structure is concerned, they are roughly equivalent. Otherwise it will soon become necessary to make further subdivisions. We have limited the standardization of the production factors to the following 17 items:

(*a*) Labour (administrative worker; technologist during normal working hours and during evening, night and weekend duties; chief technologist).

(*b*) Analytical instruments (several important instruments separately; and the rest in smaller groups).

(*c*) Raw-materials (four chemical reagent groups arranged according to their prices; control and calibration materials; glass tubes, pipettes, tubing etc.; printer paper, lint, request forms etc.)

In this study the cost for the use of the computer system has been provisionally included in the overhead. This will be described in detail in a latter study. The production quantities of the computer system, for example a system second, must therefore be precisely defined.

It is now important to establish a physical unit for the production factors. For the factors labour and analytical instruments, the time unit, minute, has been chosen for the time spent on a test. For the chemical reagent groups one must choose between a volume or weight unit. The millilitre has been selected as the physical unit here, and so reagents which are supplied in solid form must be converted into units of volume. For the control and calibration materials the unit millilitre is also used. For the collective production factors 'glass' and 'paper' the choice has fallen on a further to be defined glass unit and paper size A4, respectively. For all these units a price is determined. The labour costs are deduced from the salaries of the personnel concerned; the instrument cost consists of the depreciation of the investment and the cost for maintenance. The prices for the chemical reagent groups 1 to 4 are divided into price categories per test: less than fl 0·25; between fl 0·25 and 1·00; between fl 1·00 and 4·00; more than fl 4·00. The price of the glass unit is deduced from the prices of the various components and their weighted use contribution per test.

The production quantity for the various test types is obtained from the number of tests requested per year. It is important to note that the number of tests carried out is larger than the number requested. Control and calibration samples also employ production factors (and therefore costs) as do patient samples of which, for reasons of quality control, a part will be rejected and repeated. Therefore, in order to determine the production figures, a correction factor to raise the production quantity must be introduced for each analytical test. Further, the technical coefficients per activity must be determined for every production factor. In other words, per activity the amount of each production factor (labour, analytical instrument and raw materials) applied for each test, must be examined. In the construction of the cost structure model this action requires the most effort because of the very detailed data necessary. An example of the matrix produced for activities and production factors is shown in table 1. The example concerns a fictitious laboratory where the activities are grouped as follows:

(*a*) Instrumental, multichannel analysis system (180 000 samples in 1984).

(*b*) Instrumental, discrete value orientated analysis system for emergency tests during evening, night and weekend duties (30 000 tests in 1984).

(*c*) Manual tests (50 000 in 1984).

(*d*) Extensive manual tests (1000 in 1984).

The technical coefficients in the table should be read as follows (as an example activity the Manual tests is used): on average per test 4·70 min labour (administration worker 1·50, technologist 3·00 and chief technologist 0·20 min) is necessary, whereby the cost per min is entered in the column price per unit. These prices are deduced from the salaries of the personnel concerned and from the available personnel capacities (40 h work week minus absence for reasons such as holidays, illness, meetings and preventive service; briefly the time which is spent nonproductively on analytical tests). As a result the normal capacity of the production factor personnel remains. Per subgroup of the personnel category the normal capacity can differ. The high cost per minute for the chief technologist is the result of the assumption that he or she only spends 25% of the work capacity on the production of test results.

A low priced analytical instrument has been used for this test (colorimeter read-out) so the technical coefficient is low and can be disregarded. For the results under (*a*) and (*b*) the instrument capacity has naturally been established via the definition of the normal capacity, and the price per unit of the instrumental production factor has been deduced from this.

From the raw materials 0·65 ml chemical reagent from group 3 and 1·30 ml control and calibration materials were used (the technical coefficient for paper is too small to be mentioned). The production of all requested tests totalled 50 000 in 1984, where an average correction factor of 2·20 was applied (compensation for test/patient samples which had to be repeated; extra test and calibration materials). The calculations can now be carried out:

(*a*) All technical coefficients from each activity are multiplied by the corresponding correction factor with the exception of the technical coefficients for administrative worker and chief technologist, because these factors are practically solely dependent on the number of tests requested. A matrix of activities and production factors with technical coefficients per test requested is then produced.

(*b*) Further, the cost distribution table is determined in physical quantities by multiplying all elements for each activity by the production quantity (number of tests in 1984).

(*c*) Finally, the multiplication of the elements from the ensuing matrix with the corresponding prices per unit results in a cost distribution table in monetary units (in this example in Dutch currency). This cost distribution table, orginating from the data in the example, is illustrated in table 2.

From the available data two important surveys can be deduced:

(1) The total usage of the production factors in the production program for a (sub-)laboratory is determined, after which the total cost is established by the addition of a charge for the overhead cost, see table 4 (indirect labour expenses, housing facilities etc.). It is also possible, via personnel and instrumental capacity, to calculate the capacity used of the corresponding production factors, see table 3.

(2) The total cost of each activity is obtained by the summation of all elements in the cost distribution table of monetary units, for the corresponding activity. These are then the directly allocatable costs for all tests in 1984. The cost price per test is obtained when these costs are divided by the production quantities in 1984. This cost price must then be raised with a charge for the overhead cost, see table 4.

As far as the indrect costs (overhead) are concerned there is provisionally chosen for a general charge. After a separate investigation, these costs will be more specifically allocated.

All these calculations can be performed very quickly on a microcomputer with a 'spreadsheet' programme, for example VisiCalc, Multiplan and Lotus 1-2-3. The results of modifications in the production program in the laboratory (such as: replacement of analytical instruments, modification in staffing, changing reagents etc.), which give rise to different technical coefficients and prices per unit, are easily calculated. With the aid of this generally applicable cost structure model, every laboratory is capable of examining the influence of management decisions and proposals on the cost price of clinical chemistry investigations.

### Several examples of management decisions

Thanks to the cost structure model we have been able to form a deeper insight into the cost build-up of clinical chemistry investigation in our laboratory. With the model it is possible to test management proposals, which increases the quality level of the management decisions. Actually the model makes an integral judgement over all the direct (and indirect) cost. How tempting the dealers of analytical instruments and chemical reagents may make their offers sound (through placing favourable cost elements in the foreground and concealing or underestimating the less auspicious factors), in the application of this cost structure model all cost elements are taken into consideration. It must be emphasized that the model calculates the financial consequences of decisions. Other consequences, for example the quality of service (such as turn-around time and quality of the requested tests), can be taken into consideration. To realize this, time and/or quality must be incorporated into the model in the form of technical coefficients or activities produced, for example different activities according to differences in quality or time. Examples of management decisions which, with the aid of the cost structure model, may be judged on their financial consequences, are:

(i) Substitution of analytical instruments, where the production method changes (technical coefficients), for example substituting a multichannel analysis

*Table 1. Matrix of activities and production factors with technical coefficients in physical units for a fictitious laboratory. Activities A, B, C and D are instrumental, instrumental emergency, manual tests and extensive manual tests.*

| Production factor | Activity | *A* | *B* | *C* | *D* | Price per unit in Dutch fl. |
|---|---|---|---|---|---|---|
| | Administration worker | 0·90 | | 1·50 | | 0·54 |
| | Technologist | 1·10 | | 3·00 | 61·00 | 0·56 |
| | Technologist on duty | | 2·70 | | | 0·61 |
| | Chief technologist | 0·10 | | 0·20 | 0·20 | 2·31 |
| | Instrument A | 0·40 | | | | 1·72 |
| | Instrument B | | 0·67 | | | 0·26 |
| | Chemical reagents | | | | | |
| | group 1 | | 0·09 | | | 7·86 |
| | group 2 | | 0·91 | | | 2·54 |
| | group 3 | 1·78 | | 0·65 | 1·40 | 0·46 |
| | group 4 | 27·00 | | | | 0·02 |
| | Control and calibration materials | 0·28 | | 1·30 | | 0·78 |
| | Glass etc. | 7·50 | 0·51 | 0·40 | | 0·14 |
| | Paper etc. | 1·50 | 2·00 | | | 0·02 |
| | Production 1984 | 180 000 | 30 000 | 50 000 | 1 000 | |
| | Raise factor | 1·59 | 1·35 | 2·20 | 1·10 | |

*Table 2. Cost distribution table in Dutch fl. for a fictitious laboratory originating from the data set in table 1. Activities A, B, C and D are respectively instrumental, instrumental emergency, manual tests, extensive manual tests.*

| Production factor | Activity | *A* | *B* | *C* | *D* |
|---|---|---|---|---|---|
| | Administration worker | 87 480 | | 40 500 | |
| | Technologist | 176 400 | | 184 800 | 37 576 |
| | Technologist on duty | | 66 795 | | |
| | Chief technologist | 41 580 | | 23 100 | 462 |
| | Instrument A | 198 144 | | | |
| | Instrument B | | 7 020 | | |
| | Chemical reagents | | | | |
| | group 1 | | 28 296 | | |
| | group 2 | | 93 726 | | |
| | group 3 | 234 324 | | 32 890 | 709 |
| | group 4 | 154 548 | | | |
| | Control and calibration materials | 63 180 | | 11 540 | |
| | Glass etc. | 300 636 | 2 898 | 6 160 | |
| | Paper etc. | 8 604 | 1 620 | | |
| | Total | 1 264 896 | 200 355 | 298 990 | 38 747 |

system, which is working according to the continuous flow principle, for one or more analytical instruments working separately, each with a small analytical capacity, or vice versa.

(ii) Mechanization of manual analysis.

(iii) Alterations in salaries and prices of the production factors, for example for a new year.

(iv) The influence of shorter working hours on the capacity and occupancy of the staffing production factors.

(v) Alterations in the production (number of tests) as an increase or decrease, and the influence that it has on the total cost of the laboratory.

In all these situations, the technical coefficients and/or prices per unit must be changed in the original matrix of activities and production factors. The spreadsheet program will then automatically repeat all the calculations. The ensuing results can then be compared to the initial situation. The differences can be quickly interpreted as favourable or unfavourable to, for example the exploitation cost, staffing and instrumental capacity.

*Table 3. The use of production factors and the cost sorts for a fictitious laboratory, originating from the data set in table 1. For the overhead and capacity arbitrary data has been used.*

| Production factor | Expenditure h | fl. | | Capacity (h) | Occupancy (%) |
|---|---|---|---|---|---|
| Administration worker | 3 950 | 127 980 | (6%) | 4 500 | 88 |
| Technologist | 11 868 | 398 776 | (19%) | 14 500 | 82 |
| Technologist on duty | 1 825 | 66 795 | (3%) | 3 200 | 57 |
| Chief technologist | 470 | 65 142 | (3%) | 700 | 67 |
| Instrument A | 1 920 | 198 144 | (9%) | 200 | 87 |
| Instrument B | 450 | 7 020 | (<1%) | 4 400 | 10 |
| Chemical reagents | | | | | |
| group 1 | | 28 296 | (1%) | | |
| group 2 | | 93 726 | (4%) | | |
| group 3 | | 267 923 | (13%) | | |
| group 4 | | 154 548 | (7%) | | |
| Control and calibration materials | | 74 720 | (3%) | | |
| Glass etc | | 309 694 | (14%) | | |
| Paper etc. | | 10 224 | (1%) | | |
| Subtotal | | 1 802 988 | (84%) | | |
| Overhead | | 335 791 | (16%) | | |
| Total | | 2 138 799 | (100%) | | |

*Table 4. The total cost for each activity in fl., which means the cost price per test or sample, exclusive of additional charge for the overhead, for a fictitious laboratory, originating from the data set in table 1.*

| Activity | Total | Cost price |
|---|---|---|
| *A* Instrumental | 1 264 896 | 7·03 |
| *B* Instrumental emergency | 200 355 | 6·68 |
| *C* Manual tests | 298 990 | 5·98 |
| *D* Extensive manual tests | 38 747 | 38·75 |
| Subtotal | 1 802 988 | |
| Overhead | 335 791 | |
| Total | 2 138 799 | |

Apart from this the cost structure model is also capable of visualizing whether the mechanization of manual tests under certain conditions is financially justifiable and indicating which the most suitable type of analytical instrument is under these circumstances.

### List of definitions

*Activity*: A description of a technical transformation process that for certain reasons can be considered as a unit and that results in the production of final products or intermediate products.

*Capacity*: A certain (maximum) amount of a production factor available per time unit.

*Cost*: Aggregate of production factors used multiplied by their prices per unit to produce a certain production quantity.

*Cost centre*: An organization unit, in which all cost, having reference to certain activities, are specified. See also activity.

*Cost sort*: A collection of similar costs which form a basis element for the build-up of a cost centre.

*Final product*: The output of an activity which is sold by the organization and where the revenues are directly related. See also intermediate product.

*Intermediate product*: Intermediates or intermediate products are the output of these activities that are not sold by the organization but which are necessary to produce the final products.

*Matrix*: A relationship specification between vectors.

*Normal production quantity*: A certain production quantity used for the allocation of fixed cost.

*Occupancy*: Indicates which portion of the available capacity of production factors (staffing and instruments) is utilized.

*Production quantity*: Number of units of an activity produced in a certain time period.

*Spreadsheet programme*: A calculation programme with which, via an electronic work-sheet consisting of rows and columns, calculations can be carried out automatically.

*Technical coefficient*: Amount of a production factor which must be used in order that a unit of an activity can be produced.

*Vector*: A set of variables.

### List of abbreviations

$A$ = matrix of technical coefficients.

$a(i,j)$ = amount of production factor $i$ which is used in order to realize a unit of $g(j)$; the technical coefficient.

$B$ = matrix of production factors used per cost centre or activity ($j$) to produce $g(j)$.

$b$ = vector of $i$ production factors used (physical cost sorts).

$D$ = vector of percentages.

$E$ = unit matrix.

$F$ = matrix of percentages specifying the deliveries between cost centres.

$G$ = diagonal matrix of $g$.

$g(j)$ = number of items of the activity $j$ produced (production quantity).

$k$ = vector of cost sorts.

$K(j)$ = cost of the production of $g(j)$.

$kp$ = cost price.

$kr$ = vector of directly imputed costs.

$KS$ = matrix of cost sorts distributed over the cost centres (cost distribution table).

$Kt$ = total costs.

$Kt(j)$ = total of cost centre $j$.

$Kv$ = fixed cost of the production of $g(j)$.

$p$ = vector of prices of production factors.

$p(i)$ = price of the production factor $i$ used.

$x(i)$ = amount of the production factor $i$ used in order to produce the production quantity $g(j)$.

### Appendix

In the production centres method the cost calculation takes place as follows:

Take cost sort 1, for example the salaries of a group of staffing and divide this over the various cost centres,

$$k(1) = d(1)k(1) + d(2)k(1) + \dots d(m)k(1) \quad (4)$$

or

$$0 = d(1)k(1) + d(2)k(1) + \dots d(m)k(1) - K(1) \quad (5)$$

where:

$k(1)$ = total of cost sort 1.

$d(j)$ = the percentage of cost sort 1 which is imputed to cost centres m ($j$ = 1 to $m$), $0 < d(j) < 1$ and SUM $d(j) = 1$.

The directly imputed cost per cost centre –*kr*– are:

$$k.D = kr \quad (6)$$

All the directly imputed cost of the intermediate cost centres are the indirect cost. In the production centres method the indirect cost are allocated to the cost centres of the final products as follows:

$$kt(1) = kr(1)$$

$$kt(2) = kr(2) + f(21)kt(1)$$

$$kt(m) = kr(m) + f(m1)kt(1) + \dots f(mm)kt(m) \quad (8)$$

where:

$kt(j)$ = total cost centre $j$.
$f(jo)$ = percentage of allocated cost of cost centre $kr(j)$ which is allocated to cost centre $kr(o)$.

These equations can also be written as follows:

$$kr(1) = kt(1)$$

$$kr(2) = kt(2) - f(21)kt(1)$$

$$kr(m) = kt(m) - f(m1)kt(1) - \dots f(mm)kt(m) \quad (9)$$

or using matrix notation as:

$$kr = (E - F) . kt \quad (10)$$

### Acknowledgements

The authors wish to thank to E. E. Ligeon, Dr F. A. J. Muskiet, M. Volmer and Dr B. G. Wolthers, for their kind assistance with the cost structure model.

### References

1. Leijnse, B., den boer, N. C. and van der Heiden, C. Congress Abstracts: 'Kostenbeheersing in de Klinische Chemie' (The Hague, The Netherlands, 1984).
2. Cooper-Lybrand and Associates Ltd, *Procedure for Determining Test Costs in Pathology Laboratories* (Department of Health and Social Security, London, 1976).
3. Stilwell, J., *Journal of Clinical Pathology*, **34** (1981), 589.
4. Broughton, P. M. C. and Hogan, T. C., *Annals of Clinical Biochemistry*, **18** (1981), 330.
5. Krieg, A. F., Israel, M. and Fink, R. *et al.*, *American Journal of Clinical Pathology*, **69** (1978), 525.
6. Worth, H. G. L., *Journal of Automatic Chemistry*, **2** (1980), 125.
7. Haeckel, R. and Weirich, A., *GIT Lab. Med.*, **5** (1982), 199.
8. Lo, J. S., Pringle, P. F., Wigzell, K. F. *et al.*, *Clinical Biochemistry*, **10** (1977), 164.
9. Gibitz, H. J., *Journal of Automatic Chemistry*, **5** (1983), 79.
10. Westlake, G. E., *Journal of Automatic Chemistry*, **5** (1983), 75.
11. Haeckel, R., *Journal of Automatic Chemistry*, **5** (1983), 68.
12. Barclay, J. E., *Journal of Automatic Chemistry*, **5** (1983), 71.
13. de Vries, T. 'Het Klinisch Chemisch Laboratorium in Economisch Perspectief' (Thesis, Erasmus University Rotterdam, The Netherlands, 1974).
14. Leijten, J. F., van der Geer, F., Scholten, M. N. M. and Goldscmidt, H. M. J., *Annals of Clinical Biochemistry*, **21** (1984), 109.
15. Bosman, A. and Bouma, J. L., Cost accounting, planning and budgeting. *Quantitative Methods in Budgeting*, ed. Tilanus, C. B. (Martinus Nijhoff, Leiden, The Netherlands, 1976).
16. van Halem, C., Zevenbergen, L., van Dalen, F. J. and Hugenholtz, P. G. 'Kosteninformatiesysteem voor een Hartcentrum'. *Hart Bulletin*, **15** (1984), 212.

